# Diagnostic accuracy of cell-free DNA-based non-invasive prenatal testing for fetal aneuploidies: a systematic review

**DOI:** 10.3389/fgene.2026.1886738

**Published:** 2026-08-18

**Authors:** Sharif Alhajlah

**Affiliations:** Department of Medical Laboratories, College of Applied Medical Sciences, Shaqra University, Shaqra, Saudi Arabia

**Keywords:** cell-free DNA (cfDNA), fetal aneuploidy, non-invasive prenatal testing (NIPT), trisomy 13 (T13), trisomy 18 (T18), trisomy 21 (T21)

## Abstract

**Background:**

Through a more precise and safer means of diagnosing foetal aneuploidies, non-invasive prenatal testing (NIPT) with cell-free DNA (cfDNA) has revolutionized prenatal screening and removed the necessity of invasive diagnostic procedures. The aim of the systematic review was to determine the limits of, diagnostic accuracies, and clinical value of cfDNA-based NIPT in different foetal aneuploidies.

**Methods:**

We conducted a systematic review of studies assessing the diagnostic performance of cfDNA-based NIPT for detecting Trisomy 21 (T21), Trisomy 18 (T18), Trisomy 13 (T13), sex chromosome aneuploidies (SCA), and copy number variations (CNVs)The studies included were prospective, retrospective, or diagnostic accuracy studies that compared cfDNA results with invasive prenatal diagnostic tests, including amniocentesis, CVS, karyotyping, and chromosomal microarray analysis (CMA).

**Results:**

The number of studies reviewed was 20, and it involved a total of more than 67,500 individuals. NIPT utilizing cfDNA has proven sensitive and specific in T21, T18, and T13 and an excellent accuracy level in most risk populations. In the case of T21 there was 100% sensitivity and 100% specificity of cfDNA in various studies. For Trisomy 18 the sensitivity was between 92.3% to 100% with a high mean specificity of 99.6%–100%. The sensitivity was also good, with Trisomy 13 diagnostic performance, as it lies between 78.6% and 100% and a specificity of 99.8 to 100 percent. However, SCA and CNVs showed more variable performance with a sensitivity of between 75 to 100 per cent in SCA and between 53.6 and 97.7 per cent, respectively. The presence of discordant outcomes, particularly in relation to SCA and microdeletions, was a problem area and underscored the importance of genetic counseling and standardized procedures.

**Conclusion:**

cfDNA-based NIPT is a highly effective and non-invasive tool for screening common trisomies but has limitations in detecting SCA and CNVs. While the screening’s integration into clinical practice is beneficial, it is essential to address the limitations of discordant results and the psychological impacts on patients.

**Systematic Review Registration:**

https://www.crd.york.ac.uk/PROSPERO/view/CRD420261277611, identifier CRD420261277611.

## 1 Introduction

By offering a safer and more accurate screening method for the detection of foetal aneuploidy, non-invasive prenatal testing (NIPT) based on cell-free DNA (cfDNA) transformed prenatal care and greatly decreased the need for and risks associated with invasive diagnostic procedures like amniocentesis and chorionic villus sampling (CVS) (Moufarrej et al., 2023). This technological advancement was very helpful in the detection of the common foetal aneuploidies such as Trisomy 21 (T21), Trisomy 18 (T18), and Trisomy 13 (T13) and produced a better result compared to the conventional methods using serum-based screening in both high-risk and low-risk populations (Hardisty et al., 2016). These advances transform cfDNA into an ideal first-line screening tool in most clinical settings with its sensitivity and specificity to identify common trisomies being previously unheard of and a significant reduction in the number of risks associated with the procedure.

Early validation studies established the clinical utility of cfDNA-based NIPT as a highly accurate screening method for common fetal aneuploidies. In a landmark study, Chiu et al. demonstrated the feasibility of massively parallel sequencing of maternal plasma DNA for the non-invasive detection of fetal trisomy 21, providing a foundation for the widespWHEREread clinical adoption of cfDNA-based screening (Chiu et al., 2011). Since then, substantial technological advances have improved sequencing platforms, analytical algorithms, and overall test performance, facilitating broader implementation across diverse obstetric populations. More recently, comprehensive reviews have summarized the evolution of cfDNA technologies and highlighted ongoing challenges related to sex chromosome aneuploidies, copy number variations, and the interpretation of discordant results, emphasizing the need for continued evaluation of clinical utility and diagnostic performance (Podobnik et al., 2024).

However, though the use of cfDNA screening has been widespread in clinical practice, with its efficacy well established in the detection of common aneuploidies, the expanded use of this technology in the screening for sex chromosome aneuploidies, microdeletions, and whole genome sequencing has also added further complexity to its use in clinical practice (Hardisty et al., 2016; Yu et al., 2025). Thus, though the use of this technology has been revolutionary in the conduct of prenatal diagnostics, the expanded use of cfDNA screening has also necessitated a more critical evaluation of its utility, accuracy, and limitation in clinical practice, particularly in diverse obstetric populations (Hardisty et al., 2016; Carbone et al., 2020). This systematic review aims to provide a comprehensive evaluation of the current state of cfDNA screening, from its first validation in high-risk cohorts to its extension to the general obstetric population. It reviews the methodological advances that led to the widespread application of cfDNA testing as well as the biological limits shaping the diagnostic accuracy of cfDNA testing. Additionally, the review discusses the serious problem of discordant results, which emerged because of the identified limitations of cfDNA technology, leading to clinical ambiguity and patient anxiety (Carbone et al., 2020). Discordant results, especially for the presence of SCA and microdeletions, were ongoing issues, and standard protocols and improved genetic counseling were necessary for patient decision-making (Cohen et al., 2025; Jayashankar et al., 2023).

Furthermore, the ethical implications of the widespread adoption of cfDNA screening, especially with regard to issues of patient autonomy, equitable access, and the psychological implications of receiving false-positive or false-negative results, were discussed. As the scope of cfDNA testing increased, providing good and informed consent, along with effective genetic counseling, became more important to reduce anxiety and ensure that patients were fully informed about the implications of the results (D’ambrosio et al., 2019; Gammon et al., 2016).

The spread of cfDNA screening also required that the economic implications and cost-effectiveness of this technology be carefully considered, particularly as it was implemented into routine prenatal care in various healthcare systems. The possible cost burden, both for healthcare providers and patients, had to be balanced against the benefits of early, non-invasive detection of fetal aneuploidies (Mousavi et al., 2022). This review assessed the available evidence on the economic impact of cfDNA testing, including the affordability and accessibility of cfDNA testing in both high- and low-resource settings.

In addition, the role of regulatory frameworks and professional guidelines in the implementation and interpretation of cfDNA results was discussed to help ensure that the benefits of cfDNA screening are spread responsibly and equitably across different populations worldwide (Yu et al., 2025) further examined the clinical adoption and perception of NIPT, showing its increasing global use and acceptance as an accurate screening tool for chromosomal abnormalities, particularly among pregnant women (Bahkali et al., 2023). cfDNA-based NIPT is a screening modality and does not replace confirmatory diagnostic procedures such as amniocentesis or chorionic villus sampling (Bianchi et al., 2012).

## 2 Methodology

### 2.1 Study selection and eligibility criteria

This systematic review was performed to assess the diagnostic accuracy of cell-free DNA (cfDNA)-based prenatal screening test of fetal aneuploidy. The literature search strategy was developed using a combination of Medical Subject Headings (MeSH) and free-text terms related to cfDNA-based prenatal screening and fetal aneuploidy. The following keywords and search terms were used: “cell-free DNA,” “cfDNA,” “non-invasive prenatal testing,” “NIPT,” “non-invasive prenatal screening,” “prenatal screening,” “fetal aneuploidy,” “chromosomal abnormalities,” “trisomy 21,” “trisomy 18,” “trisomy 13,” “sex chromosome aneuploidy,” “diagnostic accuracy,” “sensitivity,” “specificity,” “positive predictive value,” and “negative predictive value.” These terms were combined using Boolean operators such as ANDand OR to ensure a comprehensive and systematic retrieval of relevant studies. The review drew evidence from twenty primary studies and reports from clinical validation.

Qualifying studies were any prospective cohort study, retrospective study and case controls in the sample of pregnant women with different clinical risk backgrounds. The high-risk pregnancies that were included in these populations were high maternal age or abnormal ultrasound, or even the general or mixed-risk obstetric populations.

The index test of concern was maternal plasma CFdNA screening done on the ground of already proven genomic sequencing technologies (Massively Parallel sequencing, MPS, Single Nucleotide Polymorphism, SNP, or targeted sequencing with specific regions of chromosomes). Only studies where the results of the cfDNA screening were confirmed by an appropriate reference standard, i.e., invasive diagnostic testing (amniocentesis or chorionic villus sampling with cytogenetic analysis) or postnatal clinical outcomes, were used, in which case of non-follow-up of invasive diagnostic testing.

Primary target conditions that were tested are common fetal autosomal trisomies (Trisomy 21, Trisomy 18 and Trisomy 13) and SCAs. Where possible, the findings of results of other chromosomal abnormalities such as copy number variants (CNVs) were also captured.

### 2.2 Data extraction and management

A pre-determined and standardized extraction framework was used to systematically extract the data out of the entire text of included studies. The variables obtained were the study characteristics (author, year of publication, country and study design) methods of testing and population characteristics (gestational age at the time of screening and maternal risk status).

In the performance assessment diagnostic process, the numerical data of the true positives, false positives, false negatives and true negatives was provided by a target condition. In cases where these values were not directly reported, they were calculated based on data on reported sensitivity, specificity and sample size. The failure rates of the test that were typically called no-call or indeterminate due to low fetal fraction or technical limitations were calculated and reported in order to put the actual performance of the screening conducted in the real world perspective.

### 2.3 Quality and risk of bias assessment

With the Quality Assessment of Diagnostic Accuracy Studies-2 (QUADAS-2) tool, the risk of bias and the quality of the methodology used in the recruited studies were evaluated. This assessment was done in four areas which were patient selection, index test, reference standard and flow and timing.

Patient selection bias was considered with special consideration since case-control designs have been demonstrated to overestimate the diagnostic accuracy in case-control designs as opposed to prospective cohort studies. The bias of variance was also checked by the assessment particularly in the case of general population research where the screen-positive results of the cfDNA screen-positive samples were usually verified by invasive diagnostic method, but the screen-negative results were mostly verified by birth outcome result or by pursuing through clinical outcome. This type of verification can lead to the overestimation of specificity and was considered during the interpretation.

The inclusion of studies was based on predefined eligibility criteria rather than recency alone, with emphasis on availability of complete diagnostic accuracy data (TP, FP, FN, TN) and appropriate reference standards. While newer large-scale studies exist, not all report extractable diagnostic accuracy parameters in a consistent format suitable for comparative synthesis. Therefore, selected studies were included to maintain methodological uniformity across analyses.

## 3 Results

### 3.1 Study selection and characteristics

**FIGURE 1:**
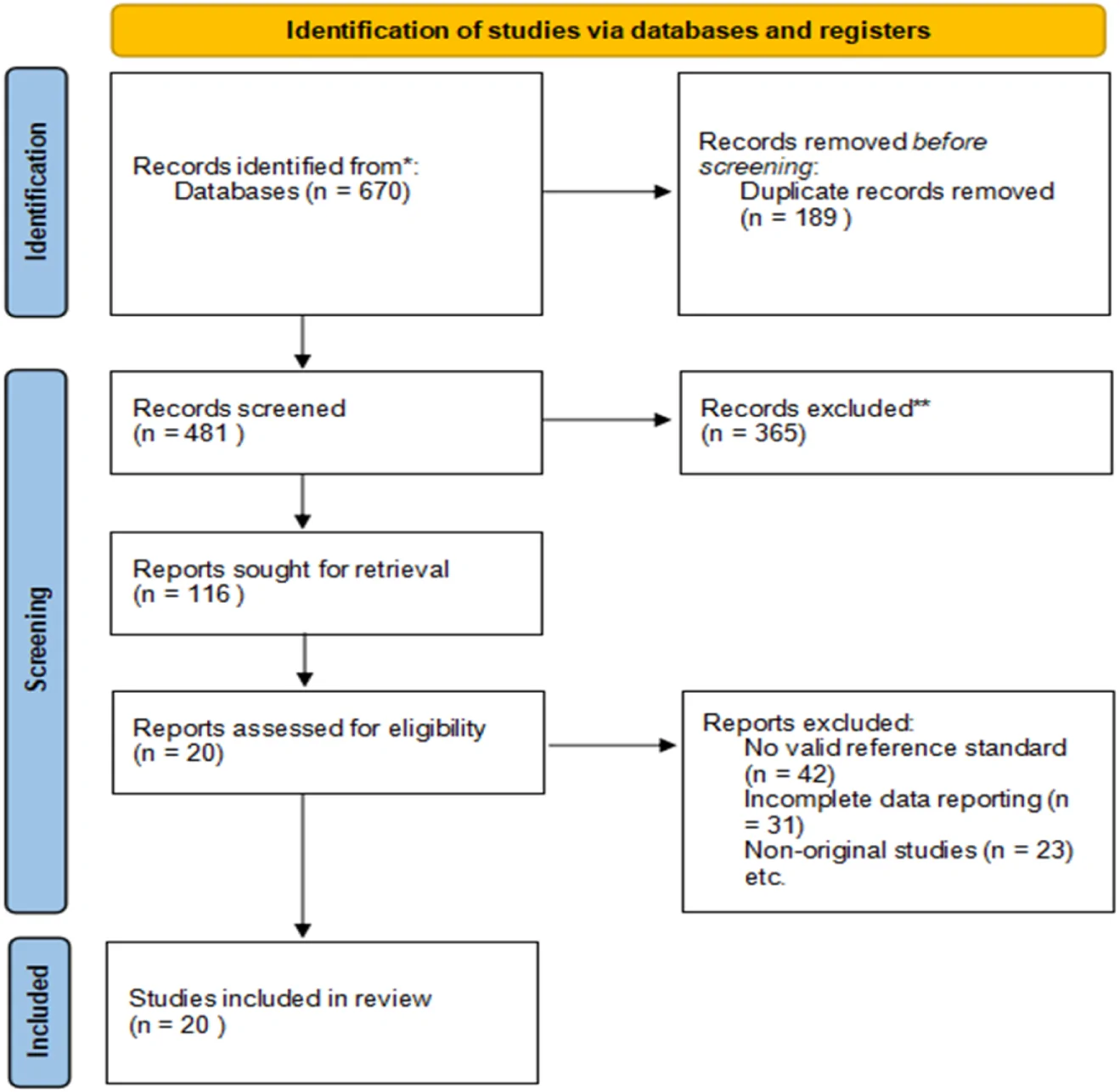
PRISMA flowchart.

A thorough search revealed 20 studies and fitted into the inclusion criterion of this systematic review. In Figure 1 (PRISMA Flowchart) the process of selecting the studies is summed up. Among the screened records (670 records) 650 records had to be ruled out due to irrelevancy, non-diagnostic reference standards or repetition of records. The final analysis included 20 studies and contained a total population size of 67,568 participants who represented various populations with the greatest number of high-risk cohorts (n = 13), mixed populations (n = 5) and general populations (n = 2). The papers were written during the years 2012 2022, the location was the United States, China, Korea, the UK, and other locations in the world.

Table 1 shows the characteristics of the study and part of its nature, including a study design, risk status of the population, gestational age, sample size, testing platform of the study on the cfDNA and the reference standard that is used in the corresponding studies. The platforms used vary with studies, but a number of studies used more than one analysis and as a result, overlap in the classification of platforms ensued. Consequently, counts through platforms are not mutually exclusive. The most common (n = 12) were karyotyping, followed by chromosomal microarray (CMA) (n = 5), and a mixture of follow-up clinical outcomes (n = 4).

**TABLE 1:** Characteristics of the included studies.

Author (Year)	Country	Study design	Population (risk status)	Gestational age	Sample size (N)	Index test (cfDNA platform)	References standard	Aneuploidy assessed
Bianchi et al. (2012)	United States	Prospective, multicenter	High-risk	10–38 weeks	532 (analysis)/2,882 (enrolled)	MPS (Illumina/Verinata)	Karyotype	T21, T18, T13, SCA
Guseh et al. (2021)	United States	Prospective cohort	High-risk (Diagnostic)	Not specified	160	Genome-wide MPS	Chromosomal microarray (CMA)	T21, T18, T13, SCA, CNVs
Han et al. (2015)	Korea	Clinical validation	High-risk and Low-risk	11–23 weeks	910	MPS	Karyotype (High-risk)/Follow-up (Low-risk)	T21, T18, T13, SCA
Hooks et al. (2014)	United States	Case-control	High-risk	>10 weeks	414	Targeted (DANSR/FORTE)	Karyotype	SCA (45,X; 47,XXX; 47,XXY)
Lai et al. (2021)	China	Prospective population-based	Mixed (General population)	12–26 weeks	83,671	MPS	Karyotype (Positive)/Follow-up (Negative)	T21, T18, T13, SCA, CNVs
Lambert-Messerlian et al. (2014)	USA/Intl	Nested case-control	High-risk and ART	10–38 weeks	740	MPS	Karyotype	T21, T18, T13
Lefkowitz et al. (2016)	United States	Retrospective, blinded	High-risk	>10 weeks	1,166	Genome-wide MPS (Sequenom)	Karyotype	T21, T18, T13, SCA, CNVs
Liao et al. (2014)	China	Retro. and prospective	Mixed	Not specified	2,275	Semiconductor sequencing (Ion Proton)	Karyotype	T21, T18, T13, SCA
Mazloom et al. (2013)	United States	Clinical validation	High-risk	10–39 weeks	1,975	MPS (Sequenom)	Karyotype	SCA
Meck et al. (2015)	United States	Retrospective review	High-risk (Referrals)	Not specified	216	Various (Commercial Labs)	Karyotype/Microarray	T21, T18, T13, SCA, Microdeletions
Nicolaides et al. (2013)	United Kingdom	Prospective	High-risk	11–13 weeks	242	SNP-based (NATUS/Natera)	CVS karyotype	T21, T18, T13, SCA, Triploidy
Pergament et al. (2014)	USA/Intl	Prospective, multicenter	High-risk and Low-risk	>9 weeks	1,051	SNP-based (Natera)	Karyotype/Neonatal exam	T21, T18, T13, SCA
Porreco et al. (2014)	United States	Prospective, multicenter	High-risk	>10 weeks	3,322	MPS	Karyotype/Birth record	T21, T18, T13, SCA
Samango-Sprouse et al. (2013)	United States	Clinical validation	High-risk	>9 weeks	187	SNP-based (NATUS/Natera)	Karyotype	SCA (45,X; 47,XXY; 47,XYY)
Song et al. (2015)	China	Pilot/Prospective	High-risk	8–12 weeks	212	MPS	Karyotype	T21, T18, T13, SCA
Wang et al. (2020)	China	Retrospective	High-risk	Second trimester	12,243	MPS	Karyotype (Positive only)	SCA
Yao et al. (2014)	China	Retrospective	Mixed	Not specified	5,950	MPS (BGI)	Karyotype/Follow-up	T21, T18, T13, SCA
Zhang et al. (2022)	China	Retrospective	Mixed (High and Low)	Not specified	68,763	MPS	Follow-up/Karyotype	T21, T18, T13, SCA
Zhu et al. (2019)	China	Prospective diagnostic trial	High-risk (Invasive indication)	2nd and 3rd trimester	802	MPS	Chromosomal microarray (CMA)	T21, T18, T13, SCA, CNVs

There was a range of different cfDNA analytical techniques used in the studies presented above, namely, massively parallel sequencing (MPS), genome-wide MPS, semiconductor sequencing, targeted sequencing techniques (DANSR/FORTE), and SNP technique. The MPS techniques were used most often in the selected studies and showed high diagnostic performance in identifying common trisomies. The SNP technique involves analyzing the genotypes of the parents and may offer additional benefits in detecting triploidy and certain sex chromosome aneuploidies. In turn, targeted sequencing is focused on particular genomic loci and can increase the effectiveness of analysis while decreasing the number of sequencing processes needed. Different sequencing chemistries, analytic techniques, determination of fetal fraction, and bioinformatics interpretation used in each of the mentioned types of analysis can serve as causes for different sensitivity, specificity, positive and negative predictive values in the studies.

### 3.2 Diagnostic performance

Diagnostic abilities of cfDNA testing in the diagnosis of fetal aneuploidies of the Trisomy 21 (T21), Trisomy 18 (T18), Trisomy 13 (T13), sex chromosome aneuploidies (SCA), and copy number variations (CNVs). A sensitivity, specificity, positive predictive value (PPV) and negative predictive value (NPV) were calculated regarding every aneuploidy and summed up across the works. It is important to note that several studies reporting 100% sensitivity were based on small sample sizes, which may limit the precision of these estimates. Such findings should be interpreted cautiously, as confidence intervals in these studies would likely be wide, reflecting underlying statistical uncertainty. Confidence intervals were not consistently reported across all included studies, and therefore could not be uniformly synthesized. As a result, diagnostic accuracy is presented descriptively, with acknowledgment of potential uncertainty in estimates derived from smaller cohorts.

For Trisomy 21, cfDNA had a sensitivity of 100% (range: 98.2%–100%) and specificity of 100% (range: 99.5%–100%) in 15 studies. This has been high and constant on various platforms, studies reported by (Bianchi et al., 2012) and (Lefkowitz et al., 2016) have reported a perfect sensitivity and specificity.

In the case of Trisomy 18 sensitivity ranges between the range of 92.3%–100%, and the mean specificity value of 99.6%–100%. An important work study by (Porreco et al., 2014) in 2014 had a sensitivity reading of 92.3% of Trisomy 18 but indicated variance in previous studies with relatively smaller sample sizes like (Zhang et al., 2022). Trisomy 13 had also high diagnostic performance with a sensitivity of 78.6%–100% and specificity of 99.8%–100%. Notably (Guseh et al., 2021), discovered 100% specificity in Trisomy 13 and lower sensitivity in certain cohorts (Mazloom et al., 2013).

Performance for sex chromosome aneuploidies (SCA) was more variable. The sensitivity ranged between 75%-100% and specificity was always at 99.4%–100%. Study by (Wang et al., 2020) reported the issues in detection of Monosomy X with a sensitivity of up to 21.4 and (Nicolaides et al., 2013) reported the 100% accuracy of SCA.

In the case of copy number variations (CNVs), the studies have shown the broader range of sensitivity (53.6%–97.7%) and strong specificity (>99%), and the sensitivity level of 97.7% in detecting CNVs, but with the limitation of the known method of detecting gene abnormalities (Table 2).

**TABLE 2:** Diagnostic performance (sensitivity, specificity, and predictive values).

Author (Year)	Aneuploidy	TP	FP	FN	TN	Sensitivity (%)	Specificity (%)	PPV (%)	NPV (%)
Bianchi et al. (201)	Trisomy 21	89	0	0	443	100	100	100	100
	Trisomy 18	35	0	1	496	97.2	100	100	99.8
	Trisomy 13	11	0	3	518	78.6	100	100	99.4
	Monosomy X	15	1	1	515	93.8	99.8	93.8	99.8
Guseh et al. (2021)	Common (T21/18/13)	21	0	0	139	100	100	100	100
	SCA	3	2	1	154	75	98.7	60	99.4
Han et al. (2015)	Trisomy 21	4	0	0	906	100	100	100	100
Hooks et al. (2014)	Monosomy X	26	2	1	378	96.3	99.5	92.9	99.7
	47,XXX	1	2	0	380	100	99.5	33.3	100
	47,XXY	6	0	0	380	100	100	100	100
Lai et al. (2021)	Trisomy 21	330	38	3	82,140	99.1	99.95	89.7	>99.9
	Trisomy 18	84	16	1	82,410	98.8	99.98	84	>99.9
	Trisomy 13	30	27	0	82,454	100	99.97	52.6	100
Lambert-Messerlian et al. (2014)	Trisomy 21 (Natural)	62	0	1	599	98.4	100	100	99.8
	Trisomy 21 (ART)	10	0	0	33	100	100	100	100
Lefkowitz et al. (2016)	Trisomy 21	85	0	0	1,081	100	100	>99.9	100
	Trisomy 18	27	0	0	1,139	100	100	>99.9	100
	Trisomy 13	15	0	0	1,151	100	100	>99.9	100
Liao et al. (2014)	Trisomy 21	55	3	0	457	99.9	99.5	94.8	100
	Trisomy 18	16	4	0	495	100	99.2	80	100
Mazloom et al. (2013)	Monosomy X	17	1	1	371	94.4	99.7	94.4	99.7
Meck et al. (2015)	Trisomy 21	92	7	-	-	-	-	93	-
	Trisomy 18	14	10	-	-	-	-	58	-
	Trisomy 13	5	6	-	-	-	-	45	-
Nicolaides et al. (2013)	Trisomy 21	25	0	0	204	100	100	100	100
Pergament et al. (2014)	Trisomy 21	58	0	0	905	100	100	100	100
	Trisomy 18	24	1	1	938	96	99.9	96	99.9
	Trisomy 13	12	0	0	953	100	100	100	100
Porreco et al. (2014)	Trisomy 21	137	3	0	3,182	100	99.9	97.9	100
	Trisomy 18	36	0	3	3,283	92.3	100	100	99.9
	Trisomy 13	14	0	2	3,306	87.5	100	100	99.9
Samango-Sprouse et al. (2013)	Monosomy X	11	0	1	172	91.7	100	100	99.4
Song et al. (2015)	Trisomy 21	2	0	0	176	100	100	100	100
	SCA (Combined)	0	1	2	175	0	99.4	0	98.9
Wang et al. (2020)	Monosomy X	3	11	-	-	-	-	21.4	-
	47,XXY	10	1	-	-	-	-	90.9	-
Yao et al. (2014)	Trisomy 21	31	0	0	5,919	100	100	100	100
	SCA (Combined)	13	11	0	5,926	100	99.8	54.2	100
Zhang et al. (2022)	Trisomy 21	107	57	2	68,597	98.2	99.9	65.2	>99.9
	Trisomy 18	25	45	1	68,692	96.2	99.9	35.7	>99.9
	Trisomy 13	7	31	0	68,725	100	99.9	18.4	100
Zhu et al. (2019)	Aneuploidy (T21/18/13)	26	5	1	770	96.3	99.3	83.9	99.9

An important but often underreported aspect of cfDNA testing is the rate of no-call or indeterminate results, frequently associated with low fetal fraction or technical limitations. High no-call rates can introduce bias in reported diagnostic accuracy, as such cases are often excluded from final analysis, potentially leading to overestimation of sensitivity and specificity. Therefore, interpretation of diagnostic performance across studies should consider the impact of test failure rates on real-world applicability.

### 3.3 Subgroup analysis by population risk

A population risk subgroup analysis. All the sensitivity and specificity were high in relation to high-risk pregnancies as compared to general/mixed populations. The sensitivity ranged between 87.5% and 100% and the specificity ranged between 99.5% and 100% in the high-risk populations. This is opposed to that of the general population where sensitivity was 96.2%–100% and specificity was even higher, 99.9%–100%.

The high accuracy of diagnoses in the high-risk pregnancies is likely to be attributed to the increased prevalence of the aneuploidies in the cohorts. Conversely, there is a slight deviation in people in general due to the reduced occurrence of fetal aneuploidies, and the freedom of false positives in the less risk groups (Table 3).

**TABLE 3:** Subgroup analysis by population type.

Subgroup	No. of studies	Sensitivity range (%)	Specificity range (%)
High-risk pregnancies	13	87.5–100	99.5–100
General/Mixed population	6	96.2–100	99.9–100

### 3.4 Quality Assessment and applicability

**FIGURE 2:**
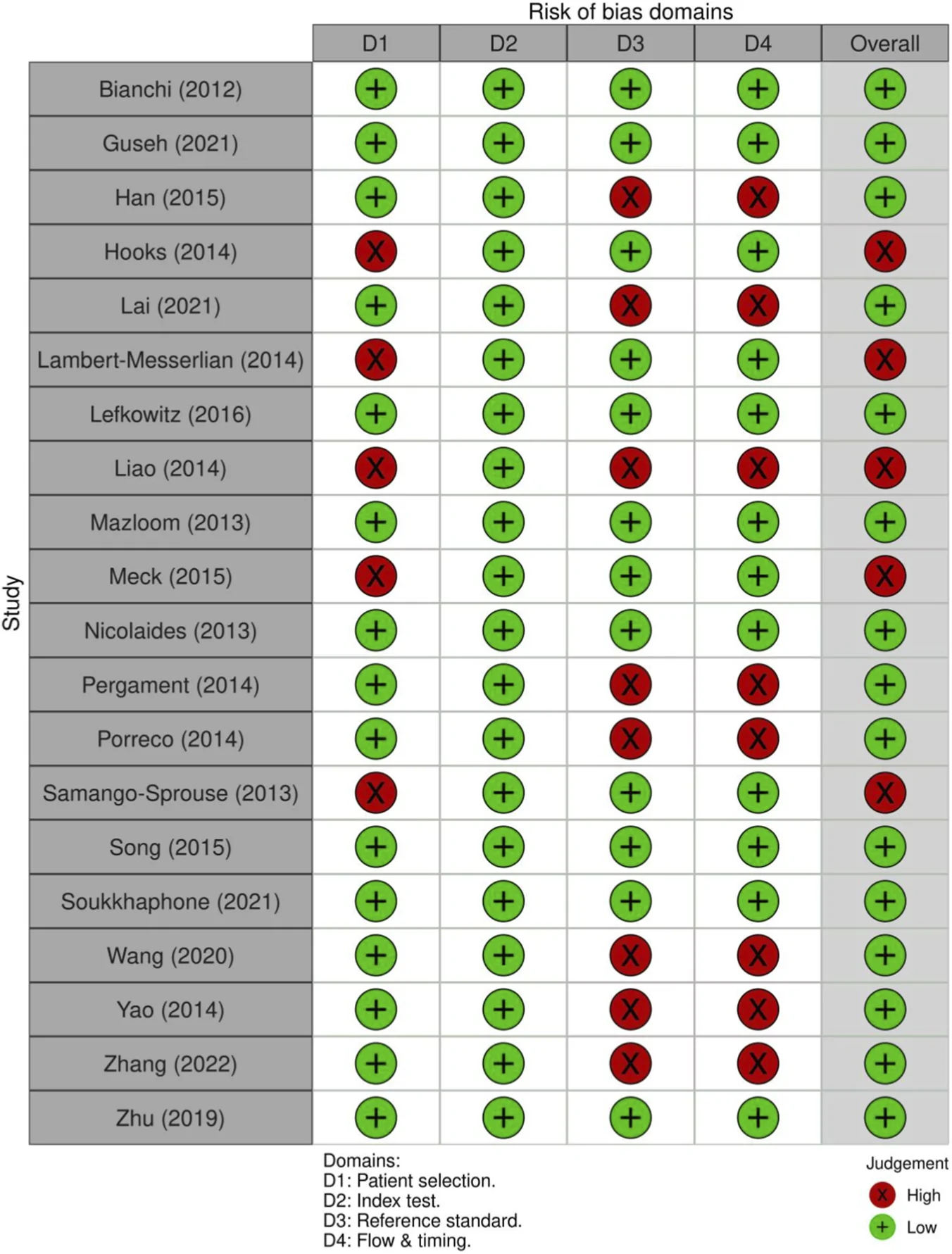
Risk of bias assessment of the included studies using the QUADAS-2 tool.

The Applicability concerns were deemed as low in most of the studies (n = 14) with regards to selection of patients, index test and reference standard and thus the findings of these studies appear to have applicability in the clinical setting. But other studies, such as those by Han et al. (2015) and Liao et al. (2014) were more open to the problem of applicability due to the mixed-risk populations and differences in gestational age and sample time (Figure 2).

### 3.5 Certainty of evidence

The certainty of evidence for each aneuploidy, based on the range of sensitivity and specificities of the different studies For Trisomy 21 the evidence certainty is high with the consistently high sensitivity (98.2%–100%) and specificity (99.5%–100%) seen in 15 studies. Likewise, there is also high rating of evidence of Trisomy 18 and Trisomy 13 which implies a good performance by the use of cfDNA in the diagnosis of these typical aneuploidies.

Evidence of SCA and CNVs is moderate, due to the increased sensitivity and specificity. In the case of CNVs, there is a low certainty of evidence due to the problem in identifying CNVs that are very rare genetic alterations and they can be complicated (Table 4).

**TABLE 4:** Certainty of evidence.

Aneuploidy	No. of studies	Sensitivity (range)	Specificity (range)	Certainty of evidence
Trisomy 21	15	98.2%–100%	99.5%–100%	High
Trisomy 18	15	92.3%–100%	99.6%–100%	High
Trisomy 13	15	78.6%–100%	99.8%–100%	Moderate
SCA (Combined)	16	75.0%–100%*	99.4%–100%	Moderate
CNVs	4	53.6%–97.7%**	>99.0%	Low

## 4 Discussion

The results show that cfDNA-based NIPT is a highly accurate screening tool in detecting T21, T18, and T13, but shows variability in screening T21 detected by cfDNA in detecting SCA and CNVs. This part will compare the diagnostic performance of the different studies in the area on the use of cfDNA, the strengths and limitations of the testing using the underlying DNA and what this implicates in clinical practice.

Beyond clinical performance metrics, the diagnostic accuracy of cfDNA-based NIPT is inherently dependent on pre-analytical and analytical laboratory processes. Factors such as cfDNA extraction efficiency, fetal fraction enrichment, and sequencing library preparation significantly influence test sensitivity and specificity. Variations in these steps can introduce technical noise, reduce fetal DNA representation, and contribute to false-positive, false-negative, or no-call results. Therefore, diagnostic accuracy should be interpreted not only as a function of test performance but also as a reflection of underlying laboratory methodologies and technological optimization (Chen et al., 2018). The yield and purity of extracted cfDNA play a critical role in determining test reliability. Inefficient extraction or contamination with maternal genomic DNA and chemical inhibitors can reduce effective fetal fraction and impair downstream sequencing accuracy. This may lead to reduced sensitivity, increased false-positive rates, and higher test failure rates. Pre-analytical variability, including sample handling, storage conditions, and extraction protocols, therefore represents a key determinant of real-world diagnostic performance (Chen et al., 2018; Soukkhaphone et al., 2021). Recent advances in microfluidic and lab-on-chip technologies have sought to address these limitations. Integrated platforms utilizing magnetic bead-based extraction and electrokinetic or electroosmotic flow mechanisms enable improved cfDNA isolation with higher purity and reduced contamination from reagents such as guanidinium salts. These innovations enhance downstream sequencing quality and contribute to more reliable detection of fetal aneuploidies, particularly in samples with low fetal fraction (Han et al., 2015; Lai et al., 2021). Another critical analytical step is DNA library preparation for next-generation sequencing. Conventional manual workflows are susceptible to pipetting variability, batch effects, and reagent inconsistencies, which can influence sequencing depth and uniformity. Automated and chip-based microfluidic platforms have been developed to standardize library preparation, reduce human error, and optimize reagent utilization, thereby improving reproducibility and diagnostic reliability in NIPT workflows (Bahkali et al., 2023; Bianchi et al., 2012).

In addition to technical factors, biological and operational challenges further influence diagnostic accuracy. Confined placental mosaicism, maternal chromosomal abnormalities, and low fetal fraction can lead to discordant results. Pre-analytical variables such as timing of sample collection and gestational age also impact cfDNA yield. Furthermore, economic considerations, including cost of sequencing platforms and throughput limitations, influence the scalability and accessibility of NIPT in different healthcare settings (Lai et al., 2021; Lambert-Messerlian et al., 2014; Lefkowitz et al., 2016). Advances in epigenetic profiling, particularly the use of differential DNA methylation patterns between maternal and fetal DNA, are also being explored to improve fetal fraction estimation and discrimination. These approaches may enhance analytical precision and reduce ambiguity in borderline cases (Lambert-Messerlian et al., 2014; Lefkowitz et al., 2016).

Trisomy 21 (T21) was invariably detected with a high sensitivity and specificity in studies. To illustrate (Bianchi et al., 2012), reported 100% sensitivity and 100% specificity of T21, using Illumina-based MPS in a sample of 532. In the same manner (Lefkowitz et al., 2016) revealed 100% sensitivity and specificity of T21 by the sequenom-genome-wide MPS in a group of 1,166 high-risk pregnant women. These results agree with previous literature to indicate that the use of cfDNA-based NIPT can be very effective in high-risk groups in detecting T21. These results were also backed by (Chen et al., 2018), which indicated that the diagnostic accuracy of cfDNA-based NIPT was evident, especially in identifying trisomies such as T21, T18 and T13, and in performing with great sensitivity and specificity across different platforms.

By contrast, the Trisomy 18 (T18) and Trisomy 13 (T13) variance showed in terms of studies arred to differ. In an analysis of a large population of 83,671 individuals (population based study) (Lai et al., 2021), determined T21 sensitivity of 99.1% and specificity of 99.95, though with a somewhat lower sensitivity of both T18 (98.8) and T13 (100%). A total of 121,276 showed 100% sensitivity in their series of 160 high-risk women by genome-wide MPS with 100% specificity of all disorders (Guseh et al., 2021). But some such as (Meck et al., 2015) and (Zhang et al., 2022) did show T18 and T13 somewhat worse (specifically) performance. Indicatively (Zhang et al., 2022), has found sensitivity of 96.2% and 99.9% specificity of T18% and 100% and 99.9% sensitivity of T13 respectively. The difference in specificity between studies could be attributed to the difference in sample size, cfDNA testing platform and reference standard (e.g., karyotyping vs. chromosomal microarray (CMA)).

These results underscore the usefulness of the cfDNA-based NIPT as a reliable method of screening typical trisomies (and, in particular, T21) in a range of different populations when using various platforms. Nevertheless, sensitivity and specificity between T18 and T13 is a bit varied with lower sensitivity in a few of the studies particularly where small sample and/or heterogenous risk groups were used.

Variability in the diagnostic accuracy of cfDNA between studies when detecting sex chromosome aneuploidies (SCA) was greater. In the cases of Monosomy X (Turner Syndrome), 47,XXX, and 47,XXY, the performance of cfDNA showed less consistency as compared to T21, T18, and T13. As an example (Samango-Sprouse et al., 2013), had sensitivity of 91.7% and specificity of 100% with Monosomy X, whereas other studies, like (Hooks et al., 2014) and (Song et al., 2015) had lower sensitivities (Hooks et al., 2014). showed 96.3 and (Song et al., 2015) showed 0 and 99.4 specificity and sensitivity respectively to Monosomy X and combined SCA respectively. It is possible that one of the reasons behind these discrepancies is the low prevalence of Monosomy X and other sex chromosome aneuploidies in the normal population leading to an increased false positive in low-risk populations. In contrast, in high-risk populations, where more frequently SCA is detected, the performance of cfDNA in detecting SCA is often better. E.g., MPS has demonstrated a sensitivity and specificity of 100 and 90.9 percent respectively to 47, XXY in a high-risk-group of individuals by (Wang et al., 2020) but merely 21.4-percent sensitivity to Monosomy X.

Differences in diagnostic performance may also be influenced by the underlying cfDNA analysis platform. Massively parallel shotgun sequencing (MPSS) and single-nucleotide polymorphism (SNP)-based approaches differ in their analytical principles. MPSS relies on quantitative counting of DNA fragments across chromosomes, whereas SNP-based methods incorporate parental genotype information to infer fetal chromosomal status. SNP-based approaches may offer advantages in detecting triploidy and distinguishing maternal from fetal contributions, while MPSS methods are widely validated and scalable across large populations. These methodological differences should be considered when interpreting variability in sensitivity and specificity across studies (Song et al., 2015; Wang et al., 2020; Yao et al., 2014; Zhang et al., 2022). The difference in detection of SCA is also dependent on the platform of cfDNA which was utilized. As an example (Nicolaides et al., 2013), and (Pergament et al., 2014) works were premised on SNP-based platforms, which can offer superior performance in SCA-detection to methods of MPS. This observation is indicative that platform-related factors, and intricacy of sex chromosome disorders, are playing a role in unequal identification of SCA.

Platform heterogeneity represents an important consideration when interpreting the overall findings of this review. Although all major cfDNA technologies demonstrated excellent performance for the detection of common trisomies, differences in sequencing chemistry, fetal fraction estimation methods, bioinformatic pipelines, and analytical algorithms may have contributed to variability in reported sensitivity, specificity, PPV, and NPV across studies (Hardisty et al., 2016; Yu et al., 2025). Because the included studies used heterogeneous platforms and reporting approaches, direct platform-specific comparisons and formal pooled analyses were not feasible. Therefore, the overall diagnostic accuracy estimates should be interpreted with caution, particularly for sex chromosome aneuploidies and copy number variations, where performance appears more platform dependent (Carbone et al., 2020; Jayashankar et al., 2023).

Copy number variations (CNVs) are a significant issue to the cfDNA based NIPT. CNV sensitivity ranged between 53.6% and 97.7 with high specificity (>99%) being reported in various studies. Genome-wide MPS (Guseh et al., 2021) was demonstrated to have 97.7% sensitivity to CNVs and genome-wide MPS (Yao et al., 2014) demonstrated 53.6% sensitivity and >99% specificity. This decreased CNV sensitivity is also in line with a past research study that indicated that cfDNA is not as sensitive to detect submicroscopic genetic changes, which tend to be challenging to detect using the cfDNA technique. There are also concerns in (Bianchi et al., 2012) regarding the variability of the precision of cfDNA-based NIPT in identifying CNVs and the issue of a more nuanced way of thinking on the topic of taking cfDNA as a screening tool against such rarer genetic changes.

This lack of CNV sensitivity, and specifically submicroscopic deletions and/or duplications, can also be attributed to the size and complexity of CNVs, which may not be sufficiently represented by the fractions of cfDNA isolated using maternal blood. In addition, there are differences in the power of the testing platforms on which these studies are conducted in their capability in identifying CNVs, with SNP-based potentially having the upper-hand in this respect compared to MPS.

The disparity between the performance of the cfDNA in high-risk and general populations indicated that there were some unique differences in sensitivity and specificity. During high-risk pregnancies sensitivity ranged between 87.5 to 100 and specificity was always at the 99.5 to 100. These findings coincide with the Trisomy 21 research by (Bianchi et al., 2012) which reported 100% sensitive and specificity of high risk pregnancies with regards to MPS. Likewise (Lai et al., 2021), and (Zhang et al., 2022) reported that the common trisomyies high-risk cohort accuracies were high.

Conversely, it showed a little more variation among the general populations with sensitivity of 96.25%–100% and specificity of 99.9%–100%. This difference in these populations can be due to the likelihood that there is more aneuploidies among the general population and therefore by the wage of a false positive when rare events occur, i.e., SCA and CNVs. The outcomes in the general population are in line with the research conducted by (Lefkowitz et al., 2016) who noted a high performance in the identification of aneuploidies in T21 although there was variation in the identification of the less prevalent aneuploidies in riskier groups. Although larger and more recent studies may better reflect current clinical performance, inclusion in this review was not restricted by publication year but by methodological compatibility. This approach ensured consistency in diagnostic accuracy reporting, though it may limit representation of the most recent technological advancements.

## 5 Implications for clinical practice

The evidence of this review supports the use of cfDNA-based NIPT as a screening approach in first-line tests in fetal aneuploidies particularly in high-risk pregnancy where the test gives high rates of accuracy in T21, T18 and T13. The invasiveness of the procedures necessitating amniocentesis and CVS tests are reduced to the lowest by the non-invasive nature of the concept that is the cfDNA testing technique is based on. In general cases, confirmation by cfDNA might be required though the sensitivity is less sensitive with SCA and the CNVs. Gestational age, fetal fraction, and status of the patient in risk should be considered when deciding whether a primary screening method should be based on the use of the cfDNA. In low-risk populations, cfDNA can be helpful in identifying high-risk pregnancies, but false positive and false negative results should be considered when interpreting cfDNA results. Emerging non-invasive approaches, such as the isolation of intact extravillous trophoblast cells from cervical or maternal samples, represent a potential alternative to cfDNA-based screening. Unlike fragmented cfDNA, these cells contain the complete fetal genome and may offer improved diagnostic resolution. Although still under investigation, such cell-based methods may help overcome some inherent limitations of cfDNA, including fragmentation and low fetal fraction.

## 6 Future research directions

Further research needs to concentrate on the improvement of the efficiency of the cfDNA in the identification of CNVs and the so-called SCA, particularly in the case of Monosomy X and in other SCAs. As well, research on the sensitivity and specificity differences based on platform, particularly between SNP-based and MPS techniques is needed. Massive studies in various cohort of individuals are also required to test the applicability of the cfDNA extension across the globe, and its efficacy in diverse ethnic and socioeconomic groups of individuals. Moreover, the psychosocial impact of false-positive and false-negative outcomes and pre-test counselling of the patients would also be important in ethically incorporating the use of cfDNA in regular clinical practice.

## 7 Limitations

However, the review faces various limitations. Firstly, there is an occurrence of verification bias due to the fact that most of the studies have verified positive findings by means of invasive techniques whereas those showing negative results were verified through follow-up observations hence underestimating specificity. Secondly, insufficient data available about low-risk population groups and lack of representation by some geographic areas could possibly undermine the external validity. Thirdly, differences in the reporting of sex chromosome abnormalities and CNVs could undermine consistency. In addition, inconsistency in the cfDNA platform, sequencing approach, and analysis algorithms used in various studies could lead to variability in reported performance measures. Finally, publication bias and use of retrospective studies could negatively influence the review findings.

The exclusion of no-call results in many studies represents a potential source of bias, as it selectively removes challenging cases from analysis. This may artificially inflate reported diagnostic accuracy and does not fully reflect real-world clinical performance, where repeat testing or invasive confirmation may be required.

## 8 Conclusion

This systematic review demonstrated that cfDNA-based NIPT is an exceptionally accurate screening method for identifying prevalent foetal aneuploidies, including T21, T18, and T13, with remarkably high sensitivity and specificity, particularly in high-risk groups. The accuracy for sex chromosomal aneuploidies (SCA) and copy number variations (CNVs) exhibited greater variability, with sensitivity ranging from 75% to 100% and 53.6%–97.7%, respectively, complicating their detection.

The possibility of inconsistent results, especially with SCA and microdeletions, may lead to clinical ambiguity and patient apprehension. Standardised methods and enhanced genetic counselling are essential for informed decision-making. Furthermore, ethical considerations, including patient autonomy and equal access, as well as the psychological ramifications of false positives and false negatives, must be meticulously addressed.

The economic implications of the cost-effectiveness and accessibility of cfDNA testing across diverse healthcare environments were examined, emphasising the necessity of meticulous incorporation into clinical practice.

## Data Availability

The original contributions presented in the study are included in the article/Supplementary Material, further inquiries can be directed to the corresponding author.
