# Supplementary material for: Diagnostic accuracy of cell-free DNA-based non-invasive prenatal testing for fetal aneuploidies: a systematic review
Source: Front Genet. 2026 Aug 18;17:1886738. doi: 10.3389/fgene.2026.1886738 (PMC13529303; doi:10.3389/fgene.2026.1886738)
Supplement: Supplementary file 1 [file DataSheet1.pdf]

NIHR | National Institute for Health and Care Research

PROSPERO
International prospective register of systematic reviews

Diagnostic Accuracy of Non-Invasive Prenatal Testing (Cell-Free DNA) for Detection of Fetal Aneuploidy

Sharif Alhajlah, Mohammad shakil Ahamad

Citation
Sharif Alhajlah, Mohammad shakil Ahamad. Diagnostic Accuracy of Non-Invasive Prenatal Testing (Cell-Free DNA) for Detection of Fetal Aneuploidy. PROSPERO 2026 CRD420261277611. Available from https://www.crd.york.ac.uk/PROSPERO/view/CRD420261277611.

REVIEW TITLE AND BASIC DETAILS

Review title
Diagnostic Accuracy of Non-Invasive Prenatal Testing (Cell-Free DNA) for Detection of Fetal Aneuploidy

Condition or domain being studied
Fetal chromosomal aneuploidies, including:Trisomy 21 (Down syndrome)Trisomy 18Trisomy 13Sex chromosome aneuploidies

Rationale for the review
This systematic review and meta-analysis aims to comprehensively evaluate and summarize the diagnostic accuracy of cfDNA-based non-invasive prenatal testing for the detection of fetal aneuploidy using invasive prenatal diagnostic methods as the reference standard. By applying standardized diagnostic test accuracy methods and exploring relevant subgroups, this review will provide up-to-date, reliable evidence to inform clinicians, policymakers, and guideline developers regarding the clinical utility and performance of NIPT in prenatal screening.

Review objectives
Primary outcomes:SensitivitySpecificityThese will be calculated for each type of fetal aneuploidy where data permit.

Additional Outcome(s)Positive predictive value (PPV)Negative predictive value (NPV)Diagnostic odds ratio (DOR)Area under the summary receiver operating characteristic curve (AUC)

Keywords

Non-invasive prenatal testing; Cell-free DNA; Trisomy 21

Country

Saudi Arabia

ELIGIBILITY CRITERIA

Population

Included

Pregnant women undergoing prenatal screening for fetal aneuploidy.Both high-risk and general obstetric populations.

Pregnant women undergoing prenatal screening for fetal aneuploidy.

Both high-risk and general obstetric populations.

Excluded

Animal studiesIn vitro or laboratory-only studiesStudies involving non-pregnant participants

Intervention(s) or exposure(s)

Included

Non-invasive prenatal testing (NIPT) using cell-free fetal DNA (cfDNA) obtained from maternal blood.Non-invasive prenatal testing (NIPT) using cell-free fetal DNA (cfDNA) obtained from maternal blood.

Excluded

Studies evaluating only conventional prenatal screening methods (e.g., serum markers, ultrasound) without cfDNA-based NIPT.

Studies assessing cfDNA for conditions other than fetal aneuploidy (e.g., monogenic disorders only).

Comparator(s) or control(s)

This review does not have any comparators

Study design

Only nonrandomized study types will be included.

Included

Diagnostic accuracy studiesProspective or retrospective cohort studiesCross-sectional studies reporting diagnostic accuracy outcomes

Excluded

Case reportsCase series without diagnostic accuracy dataReviews, systematic reviews, meta-analysesEditorials, letters, commentaries, conference abstracts without full data

Context

Prenatal screening and diagnostic settings, including both high-risk and general obstetric populations.

TIMELINE OF THE REVIEW

Date of first submission to PROSPERO
03 January 2026

Review timeline
Start date: 3 January 2026. End date: 3 June 2026.

Date of registration in PROSPERO
03 January 2026

AVAILABILITY OF FULL PROTOCOL

Availability of full protocol
A full protocol has been written but is not available because:

A full protocol is currently being prepared. It will be made available upon reasonable request from the corresponding author once finalized.

SEARCHING AND SCREENING

Search for unpublished studies
Only published studies will be sought.

Main bibliographic databases that will be searched
The main databases to be searched are *MEDLINE*, *PubMed*, *SCI - Science Citation Index* and *Scopus*.

Search language restrictions
There are no language restrictions.

Search date restrictions
There are no search date restrictions.

Other methods of identifying studies
Other studies will be identified by: *contacting authors or experts*.

Link to search strategy
A full search strategy is available in the full protocol as described in the *Availability of full protocol* section

Selection process
Studies will be screened independently by at least two people (or person/machine combination) with a process to resolve differences.

Other relevant information about searching and screening

Duplicate records will be removed prior to screening. Titles, abstracts, and full texts will be screened independently by two reviewers using predefined criteria. Disagreements will be resolved by consensus or a third reviewer. Study selection will be documented using a PRISMA flow diagram.

DATA COLLECTION PROCESS

Data extraction from published articles and reports

Data will be extracted independently by at least two people (or person/machine combination) with a process to resolve differences.

Authors will be asked to provide any required data not available in published reports.

Study risk of bias or quality assessment

Risk of bias will be assessed using: *QUADAS-2*

Data will be assessed independently by at least two people (or person/machine combination) with a process to resolve differences.

Additional information will be sought from study investigators if required information is unclear or unavailable in the study publications/reports.

Reporting bias assessment

Yes. Risk of bias due to missing or selectively reported results will be assessed as part of the overall risk of bias evaluation using the QUADAS-2 framework and by examining study reporting completeness and potential publication bias.

Certainty assessment

Yes. The certainty of evidence for the main diagnostic accuracy outcomes will be assessed using an appropriate approach for diagnostic test accuracy reviews, such as the GRADE framework adapted for diagnostic accuracy, considering risk of bias, inconsistency, indirectness, imprecision, and publication bias.

OUTCOMES TO BE ANALYSED

Main outcomes

Primary outcomes will be sensitivity and specificity of cfDNA-based non-invasive prenatal testing for detection of fetal aneuploidy compared with invasive diagnostic reference standards.

Additional outcomes

Additional outcomes will include PPV, NPV, diagnostic odds ratio, likelihood ratios, and area under the SROC curve.

PLANNED DATA SYNTHESIS

Strategy for data synthesis

Formal data synthesis is planned. Where sufficient and appropriate data are available, a meta-analysis of diagnostic accuracy will be conducted using random-effects models to pool sensitivity and specificity and to generate summary receiver operating characteristic (SROC) curves.

CURRENT REVIEW STAGE

Stage of the review at this submission

Review stage	Started	Completed
Pilot work		
Formal searching/study identification		
Screening search results against inclusion criteria		
Data extraction or receipt of IPD		
Risk of bias/quality assessment		
Data synthesis		

Review status

The review is currently planned or ongoing.

Publication of review results

Results of the review will be published in English.

REVIEW AFFILIATION, FUNDING AND PEER REVIEW

Review team members

Dr Sharif Alhajlah (review guarantor). ORCID: 0000-0002-9927-4073. Shaqra University. Saudi Arabia.

No conflict of interest declared.

Dr Mohammad shakil Ahamad. Shaqra University. Saudi Arabia.

No conflict of interest declared.

Named contact

Dr Mohammad shakil Ahamad (m.shakil@mu.edu.sa). Shaqra University. Saudi Arabia.

Review affiliation

Shaqra University

Funding source

Review has no funding and no agreed support from an academic institution and is done in authors' own time.

Peer review

There has been no peer review of this planned review.

ADDITIONAL INFORMATION

Review conflict of interest

Declared individual interests are recorded under team member details.. No additional interests are recorded for this review.

Medical Subject Headings

Noninvasive Prenatal Testing; Cell-Free Nucleic Acids; Chromosome Disorders; Trisomy 18 Syndrome

SIMILAR REVIEWS

Check for similar records already in PROSPERO

Similar records were not formally checked because this review focuses on providing updated and comprehensive diagnostic accuracy evidence incorporating recent studies, methodological advancements, and relevant subgroup analyses not consistently addressed in previous reviews.

PROSPERO version history

- Version 1.0, published 03 Jan 2026

Disclaimer

The content of this record displays the information provided by the review team. PROSPERO does not peer review registration records or endorse their content.

PROSPERO accepts and posts the information provided in good faith; responsibility for record content rests with the review team. The guarantor for this record has affirmed that the information provided is truthful and that they understand that deliberate provision of inaccurate information may be construed as scientific misconduct.

PROSPERO does not accept any liability for the content provided in this record or for its use. Readers use the information provided in this record at their own risk.

Any enquiries about the record should be referred to the named review contact
